# Premeal Low-Fat Yogurt Consumption Reduces Postprandial Inflammation and Markers of Endotoxin Exposure in Healthy Premenopausal Women in a Randomized Controlled Trial

**DOI:** 10.1093/jn/nxy046

**Published:** 2018-05-15

**Authors:** Ruisong Pei, Diana M DiMarco, Kelley K Putt, Derek A Martin, Chureeporn Chitchumroonchokchai, Richard S Bruno, Bradley W Bolling

**Affiliations:** 1 Department of Nutritional Sciences, University of Connecticut, Storrs, CT; 2 Department of Food Science, University of Wisconsin-Madison, Madison, WI; 3 Human Nutrition Program, The Ohio State University, Columbus, OH

**Keywords:** endotoxin, inflammation, obesity, postprandial, yogurt

## Abstract

**Background:**

Metabolic endotoxemia is associated with obesity and contributes to postprandial inflammation.

**Objective:**

We aimed to determine if low-fat yogurt consumption prevents postprandial inflammation and dysmetabolism in healthy women by inhibiting biomarkers of metabolic endotoxemia.

**Methods:**

Premenopausal women defined as obese and nonobese [body mass index (BMI, in kg/m^2^) 30–40 and 18.5–27, respectively, *n* = 120] were randomly assigned to consume 339 g of low-fat yogurt (YN, yogurt nonobese; YO, yogurt obese) or 324 g of soy pudding (CN, control nonobese; CO, control obese) for 9 wk (*n* = 30/group). The intervention foods each supplied 330 kcal with 3 g fat, 66 g carbohydrate, and 4–6 g protein. At weeks 0 and 9, participants ingested 226 g of yogurt or 216 g of soy pudding before a meal providing 56–60 g fat, 82 g carbohydrate, and 28–30 g protein. Plasma soluble CD14 (sCD14), lipopolysaccharide-binding protein (LBP), LPS activity, interleukin-6 (IL-6), glucose, triglyceride, and insulin were measured hourly for 4 h to assess differences in postprandial responses between groups by 2-factor ANOVA.

**Results:**

Premeal yogurt consumption prevented the postprandial decrease in sCD14 net incremental area under the curve (net iAUC) by 72% in obese individuals at week 0 (*P* = 0.0323). YN and YO had ≥40% lower net iAUC of LBP-to-sCD14 ratio and plasma IL-6 concentration than CN and CO, respectively (*P_ _*< 0.05). CO had postprandial hyperglycemia which was not evident in YO; in contrast YN had 57% less postprandial hypoglycemia than did CN (*P*-interaction = 0.0013). After 9 wk of yogurt consumption, ΔAUC of LBP-to-sCD14 ratios of YO and YN were less than half of those of the control groups (*P* = 0.0093).

**Conclusion:**

Yogurt consumption improved postprandial metabolism and biomarkers of metabolic endotoxemia in healthy premenopausal women. Premeal yogurt consumption is a feasible strategy to inhibit postprandial dysmetabolism and thus may reduce cardiometabolic risk. This trial was registered at clinicaltrials.gov as NCT01686204.

## Introduction

Postprandial inflammation is associated with an increased risk for insulin resistance and atherosclerosis (1). High-energy challenge meals have been used to assess the role of diet in preventing postprandial inflammation and metabolic dysfunction (2–4). Low-grade postprandial inflammation is usually transient and results from postprandial hyperglycemia and hyperlipidemia (1). In the postprandial state, increased glucose and free fatty acids enter the Krebs cycle, overwhelming the capacity for oxidative phosphorylation, leading to oxidative stress that further activates inflammatory pathways (1, 5).

The intestinal barrier function of obese individuals is compromised, leading to increased chronic inflammation and low-grade endotoxin exposure, the latter of which has been defined as metabolic endotoxemia (6, 7). Bacterial endotoxins traverse the intestinal barrier and induce systemic inflammation by coabsorption with dietary lipids or bacterial translocation (7, 8). LPS inflammatory signaling is mediated by binding to LPS-binding protein (LBP) and membrane-bound or soluble CD14 (sCD14) before activating the Toll-like receptor 4/myeloid differentiation factor 2 complex (7).

Dairy proteins and calcium may attenuate postprandial hyperlipidemia and hyperglycemia by insulinotropic activity, delaying gastric emptying, and decreasing fat absorption (9–11). Yogurt may also improve intestinal barrier function by modifying gut microbiota, stimulating the production of intestinal mucins, increasing secretory immunoglobulin A and antimicrobial peptide secretion, and maintaining function of tight junctions (7, 12). Consumption of low-fat yogurt for 4 wk decreased plasma endotoxin, LBP, and sCD14 in elderly individuals (13). Moreover, a recent meta-analysis of observational studies indicated a nonlinear inverse association between yogurt intake and type 2 diabetes risk (14). Therefore, we hypothesized that premeal yogurt consumption would reduce postprandial biomarkers of metabolic endotoxemia and inflammation, and improve metabolism in obese women to a greater extent than in nonobese women given the inherent intestinal barrier dysfunction associated with obesity.

## Methods

### Participants

The study protocol was approved by the Institutional Review Boards at the University of Connecticut (#H12-168) and University of Wisconsin-Madison (#2014-0669) and participants provided written consent before participating in study procedures. The study was registered at clinicaltrials.gov as NCT01686204. Apparently healthy premenopausal women aged 21–55 y were recruited from the Storrs, CT and Madison, WI areas from October 2012 to April 2015 by mass emails, flyers, and announcements in newspapers. The study site changed from CT to WI in July 2014 owing to relocation of the study’s principal investigator. After providing consent, participants completed a questionnaire that included demographic and health information. Study personnel then measured participants’ height, weight, waist circumference, and blood pressure. The study inclusion criteria included: BMI (in kg/m^2^) from 18.5 to 27 or from 30 to 40; an age of 21–55 y; stable body mass for the previous 2 mo; blood pressure <140/90 mm Hg; and willingness to avoid yogurt and probiotic-containing foods and consume the provided 339 g (12 oz) of yogurt or 324 g of soy pudding (control treatment) for the duration of the study. The exclusion criteria were: previous diagnosis of cardiovascular disease, diabetes, or arthritis; current cancer treatment, estrogen replacement therapy, or use of anti-inflammatory drugs; current weight-loss, kosher, vegetarian, or vegan diet; current smoking or use of dietary supplements; allergies to soy, egg, or milk; pregnant, lactating, or seeking to become pregnant.

### Experimental design

The postprandial experiments described here constitute the secondary and exploratory analyses of a randomized, controlled study to determine if yogurt consumption improves intestinal barrier function and chronic inflammation relative to consumption of a nondairy control food. The results of the recruitment, primary study outcome, additional secondary and exploratory outcomes, and compliance are described elsewhere (15). Briefly, consumption of low-fat yogurt for 9 wk reduced fasting markers of chronic inflammation and endotoxin exposure in premenopausal women, as indicated by lower TNF-α/soluble TNF-Receptor II and LBP/sCD14, as well as higher plasma IgM endotoxin-core antibody (15). In contrast, fasting plasma sCD14, the primary study outcome, was unchanged (15). The study was designed to detect a significant difference in fasting plasma sCD14 among *n* = 30/group, giving an 8.2% margin of error (15, 16). Postprandial sCD14 and IL-6 were original secondary outcome measures, whereas all other outcomes were exploratory. After the initial screening, 128 subjects were enrolled and randomly assigned to either the yogurt group or the soy pudding control group, in blocks of 6 according to obesity status, by the study coordinators (17). Participants were randomized upon enrollment by assigning random numbers generated by the principal investigator using Minitab 17.0 (Minitab Inc., State College, PA). To avoid confounding by probiotic or dairy consumption, the participants restricted consumption of dietary supplements, fermented foods, and limited their dairy consumption to ≤4 servings/d for 2 wk before the intervention (washout period, week –2 to week 0) and throughout the intervention. Dietary records were collected at weeks 0 and 9 and the data were reported elsewhere (15). From the beginning of week 0 to the end of week 9 (intervention period), the subjects were instructed to consume 339 g of yogurt (12 oz, providing 1.5 servings/d) or 324 g of control food/d. Participants and study personnel were not blinded to the intervention. Trial enrollment ended when ≥30 participants/group completed all aspects of the intervention.

To determine the acute and long-term effects of yogurt consumption on postprandial metabolism, 2 identical challenge meal tests were administered at the beginning (week 0) and end (week 9) of the intervention. For each test, participants were instructed to fast overnight and arrive at the study center the next morning. Fasting blood samples (0 h) were collected from the antecubital vein into evacuated tubes containing sodium heparin or EDTA (Becton, Dickinson and Company, Franklin Lakes, NJ). The participants were instructed to consume 226 g (8 oz, 1 serving/d) of yogurt or 216 g of control food immediately before the challenge meal. Blood samples were collected by venipuncture at 1, 2, 3, and 4 h postprandially. All blood samples were placed in an ice bath immediately and centrifuged (4°C, 15 min, 1500 × *g*) within 20 min of collection. Aliquots of plasma were snap-frozen in liquid nitrogen. All samples were stored at –80°C until analysis. During the postprandial phase, the subjects were asked to remain at the study center and avoid exercise.

### Composition of challenge meal and intervention foods

The challenge meal was formulated to induce metabolic dysfunction as described by others (3, 4). It was a high-fat, high-carbohydrate meal that consisted of 2 sausage, egg, and cheese sandwiches (Jimmy Dean, Peoria, IL) and 2 hash browns (64 g/patty, Mr. Dee's, Libertyville, IL). Owing to slight changes in meal composition because of product reformulation, the meal provided 56–60 g total fat (∼54% of total energy), 82 g carbohydrates (∼34% of total energy), and 28–30 g protein (∼12% of total energy), supplying a total of ∼960 kcal.

The intervention foods consisted of commercially available low-fat yogurt (Yoplait, General Mills, Inc., Minneapolis, MN) and soy pudding as control food (ZenSoy, South Hackensack, NJ) purchased from local suppliers. The soy pudding served as a nondairy control food with macronutrient and micronutrient content, total calories, and texture matched to yogurt (**Supplemental Table 1**). Soy pudding contained (mean ± SEM) 2.00 ± 0.09 mg/100 g of daidzein, 1.64 ± 0.01 mg/100 g of glycitein, and 5.34 ± 0.16 mg/100 g of genistein (**Supplemental Methods**). Therefore, the 324 g of control provided minimal isoflavones, equivalent to ∼5.0 g of soy bean (18), and 2–3 g of soy protein, much less than the 25 g of soy protein estimated to lower coronary heart disease risk (FDA 21 CFR 101.82).

### Biomarker analysis

Analysis of plasma glucose, TGs, and insulin were exploratory outcomes to evaluate postprandial dysmetabolism. Glucose was determined in sodium heparin plasma by a commercial colorimetric enzymatic assay kit of glucose oxidase (Cat # 10009582; Cayman Chemical, Ann Arbor, MI). Total TG was measured in sodium heparin plasma by a commercial enzymatic kit containing glycerol kinase and glycerol phosphate oxidase (Cat # 461-08992 and 461-09092; Wako Diagnostics, Richmond, VA). Insulin was determined in sodium heparin plasma by immunoassay (Cat # 80-INSHU-E01.1; Alpco Diagnostics, Salem, NH). IL-6 and sCD14 were measured by immunoassay in EDTA and sodium heparin plasma, respectively (IL-6, Cat # SS600B, high-sensitivity; sCD14, Cat # DC140; R&D System, Minneapolis, MN). LBP was determined in EDTA plasma by ELISA (Cat # HK315-02; Hycult Biotech, Uden, Netherlands). All the measurements were performed on a SpectraMax M2 microplate reader (Molecular Devices, Sunnyvale, CA) according to manufacturers’ instructions. LPS activity was quantified in EDTA plasma by the PyroGene Recombinant Factor C endotoxin detection system (Cat # 50-658U; Lonza Group Ltd, Allendale, NJ) (19). Samples were diluted 100-fold in endotoxin-free water in triplicate and concentrations were determined in triplicate through the use of external calibration curves of endotoxin from *Escherichia coli* 055:B55 from 0.005–5.0 endotoxin units (EU)/mL and reference control samples on the same plates. Therefore, the lowest detection threshold was 0.5 EU/mL plasma. Interassay relative SD was 4.7% for EDTA control plasma.

### Statistical analysis

All data were expressed as means ± SEMs. Statistical analysis was conducted via SAS 9.4 software (SAS Institute Inc., Cary, NC). The significance level was set at α = 0.05 for all statistical tests. The postprandial changes in biomarker AUC and net incremental AUC (iAUC, calculated as AUC – baseline × time) were calculated via the trapezoidal method. The difference between postprandial AUCs (week 9 – week 0) was used to determine the change over time in postprandial function. At week 0, the net iAUC approach was used to minimize biologically irrelevant deviation from the baseline values which would otherwise be induced by the use of the positive iAUC or AUC (20). The net iAUC particularly favors postprandial biomarkers that exhibit weak changes. To evaluate the effects of obesity status (obese compared with nonobese), dietary treatment (low-fat yogurt compared with control food), and the obesity × treatment interaction on net iAUC, AUC or ΔAUC were determined by 2-factor ANOVA (general linear model procedure—PROC GLM) as described in the Tables and Figures. If a significant interaction effect was detected, treatment effect was further tested within the obese and nonobese participants separately through the use of a post hoc *F* test (slice = obesity), where *P *< 0.05 was considered significant.

## Results

### Postprandial biomarkers of intestinal barrier function

**FIGURE 1:**
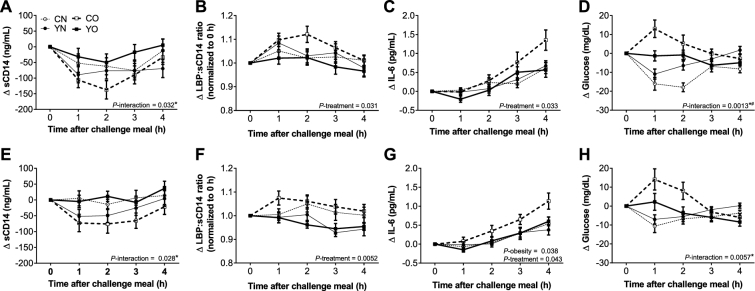
Incremental postprandial changes in plasma sCD14 (A), LBP:sCD14 ratio (B), IL-6 (C), and glucose (D) at week 0 of the intervention and in plasma sCD14 (E), LBP:sCD14 ratio (F), IL-6 (G), and glucose (H) at week 9 of the intervention in healthy obese and nonobese premenopausal women who consumed low-fat yogurt or the control food followed by the challenge meal. Data are means ± SEMs, *n* = 30. The effects of obesity status (obese compared with nonobese), dietary treatment (low-fat yogurt compared with control food), and the obesity × treatment interaction on net iAUC were determined by 2-factor ANOVA (PROC GLM). A post hoc *F* test (sliceby) was applied when *P*-interaction was <0.05. *YO different from CO, *P* < 0.05. ^#^YN different from CN, *P* < 0.05. CN, control nonobese; CO, control obese; iAUC, incremental AUC; LBP, LPS-binding protein; sCD14, soluble CD14; YN, yogurt nonobese; YO, yogurt obese; Δ, difference.

Biomarkers of endotoxin exposure were used to assess postprandial intestinal barrier function. Plasma sCD14, LBP, LBP/sCD14, and LPS activity showed dynamic postprandial changes upon the first challenge meal (**Figure 1**A–D, **Table 1**, **Supplemental Figure 1**). Premeal yogurt consumption led to 72% higher net iAUC of sCD14 in the obese group than consumption of the control snack, whereas the nonobese groups were not different between treatments (**Table 2**, *P*-interaction = 0.032). LBP/sCD14 values were significantly different between treatments (*P_ _*= 0.031), as LBP/sCD14 net iAUC was higher in the yogurt obese (YO) and yogurt nonobese (YN) groups. In contrast, LPS activity net iAUC was not different between groups (*P* = 0.69, **Supplemental Figure 2**). Furthermore, LPS activity maximum concentration (C_max_) values were not different between groups or changed by treatment (*P* > 0.05, **Supplemental Table 2**). After 9 wk of consumption of 339 g (12 oz) of yogurt or the control, sCD14 and LBP ΔAUC were not different between groups after the second challenge meal. However, at the second challenge meal, LBP/sCD14 ΔAUC (AUC_wk 9_ – AUC_wk 0_) for premeal yogurt consumption was less than the control (**Table 3**, *P* = 0.0093). The LBP/sCD14 ΔAUC was greater in nonobese individuals than obese (*P_ _*= 0.020). Postprandial LPS activity was not determined at the second challenge meal, given that yogurt consumption did not change the postprandial response of this biomarker.

**TABLE 2:** Postprandial net iAUCs of plasma biomarkers in healthy obese and nonobese premenopausal women after consuming either low-fat yogurt or control food followed by the baseline challenge meal^1^

	Week 0				Significance (*P*)^3^		
Net iAUC_0–4h_^2^	CN	YN	CO	YO	Obesity	Treatment	Interaction
sCD14 (ng/mL · h)	−197 ± 89	−277 ± 73	−350 ± 72	−96.8 ± 72.9*	0.86	0.26	0.032
LBP (μg/mL · h)	−0.26 ± 0.44	−0.95 ± 0.83	−0.04 ± 0.55	−1.76 ± 1.16	0.72	0.14	0.53
LBP:sCD14	1.23 ± 0.44	0.70 ± 0.65	2.74 ± 0.83	−0.26 ± 1.10	0.74	0.031	0.13
LPS activity (EU/mL · h)	1.04 ± 2.77	2.18 ± 3.58	0.22 ± 2.06	1.22 ± 2.04	0.74	0.69	0.98
IL-6 (pg/mL · h)	1.22 ± 0.40	0.70 ± 0.30	2.00 ± 0.63	0.61 ± 0.35	0.43	0.033	0.32
Glucose (mg/dL · h)	−44.9 ± 7.7	−19.4 ± 8.1^#^	16.7 ± 10.1	−10.8 ± 6.0*	<0.0001	0.90	0.0013
TG (mg/dL · h)	14.4 ± 34.0	29.7 ± 27.9	26.5 ± 30.9	40.4 ± 32.5	0.0027	0.60	0.14

^1^Data are means ± SEMs, *n* = 30. *Different from CO, *P* < 0.05. ^#^Different from CN, *P* < 0.05. CN, control nonobese; CO, control obese; EU, endotoxin units; iAUC, incremental AUC; LBP, LPS-binding protein; sCD14, soluble CD14; YN, yogurt nonobese; YO, yogurt obese.

^2^The net iAUC was calculated as total AUC – baseline × time.

^3^The effects of obesity status (obese compared with nonobese), dietary treatment (low-fat yogurt compared with control food), and the obesity × treatment interaction on net iAUC were determined by 2-factor ANOVA (PROC GLM). A post hoc *F* test (sliceby) was applied when *P*-interaction was <0.05 to determine treatment effects within the obese and nonobese groups.

**TABLE 1:** Comparison of postprandial plasma biomarker AUCs at week 0 in healthy obese and nonobese premenopausal women after consuming either low-fat yogurt or control food followed by the baseline challenge meal^1^

	Week 0				Significance (*P*)^2^		
AUC_0–4h_	CN	YN	CO	YO	Obesity	Treatment	Interaction
sCD14 (ng/mL · h)	5490 ± 180	5330 ± 210	5570 ± 170	5460 ± 200	0.59	0.48	0.92
LBP (μg/mL · h)	39.4 ± 3.7	36.4 ± 2.2	49.5 ± 2.9	47.4 ± 3.6	0.0012	0.42	0.90
LBP:sCD14	29.8 ± 3.0	28.3 ± 1.8	37.2 ± 2.7	36.3 ± 2.9	0.0045	0.64	0.91
IL-6 (pg/mL · h)	4.21 ± 0.40	4.45 ± 0.44	8.21 ± 0.68	8.05 ± 0.77	0.0001	0.95	0.73
Glucose (mg/dL · h)	299.5 ± 7.9	317.4 ± 8.3	375.5 ± 10.5	346.8 ± 6.7	0.0001	0.72	0.40
TG (mg/dL · h)	390.0 ± 25.7	393.8 ± 35.3	528.9 ± 39.4	540.6 ± 45.1	0.0002	0.83	0.92

^1^Data are means ± SEMs, *n* = 30. CN, control nonobese; CO, control obese; LBP, LPS-binding protein; sCD14, soluble CD14; YN, yogurt nonobese; YO, yogurt obese.

^2^The effects of obesity status (obese compared with nonobese), dietary treatment (low-fat yogurt compared with control food), and the obesity × treatment interaction on week 0 AUC were determined by 2-factor ANOVA (PROC GLM).

**TABLE 3:** Change in postprandial plasma biomarker AUC in healthy obese and nonobese premenopausal women after consuming low-fat yogurt or control food followed by challenge meals at baseline and week 9 of the intervention^1^

	Week 9 – week 0				Significance (*P*)^2^		
Δ AUC_0–4h_	CN	YN	CO	YO	Obesity	Treatment	Interaction
sCD14 (ng/mL · h)	−188 ± 155	−292 ± 130	−231 ± 106	114 ± 138	0.18	0.37	0.10
LBP (μg/mL · h)	2.48 ± 1.75	−0.55 ± 1.41	0.07 ± 1.13	−2.21 ± 1.36	0.16	0.065	0.79
LBP:sCD14	3.54 ± 1.66	1.23 ± 1.30	1.61 ± 1.05	−2.99 ± 1.16	0.020	0.0093	0.38
IL-6 (pg/mL · h)	−0.16 ± 0.67	0.17 ± 0.55	−0.25 ± 0.54	−1.19 ± 0.49	0.20	0.59	0.27
Glucose (mg/dL · h)	3.56 ± 7.17	3.29 ± 8.67	16.8 ± 12.1	2.54 ± 7.98	0.50	0.43	0.45
TG (mg/dL · h)	122 ± 14	106 ± 15	150 ± 17	184 ± 22	0.72	0.64	0.98

^1^Data are means ± SEMs, *n* = 30. CN, control nonobese; CO, control obese; LBP, LPS-binding protein; sCD14, soluble CD14; YN, yogurt nonobese; YO, yogurt obese; Δ, difference.

^2^The effects of obesity status (obese compared with nonobese), dietary treatment (low-fat yogurt compared with control food), and the obesity × treatment interaction on changes in total AUC were determined by 2-factor ANOVA (PROC GLM).

### Postprandial IL-6

IL-6 was determined as a biomarker of postprandial inflammation after the first and second challenge meals. Plasma IL-6 progressively increased after the first challenge meal (Figure 1C). Premeal yogurt consumption reduced net iAUC in obese and nonobese groups by 70% and 43%, respectively (Table 2, *P* = 0.033). Chronic yogurt consumption did not provide any additional benefit, as IL-6 ΔAUC (AUC_wk 9_ – AUC_wk 0_) was unchanged (*P* = 0.59).

**FIGURE 2:**
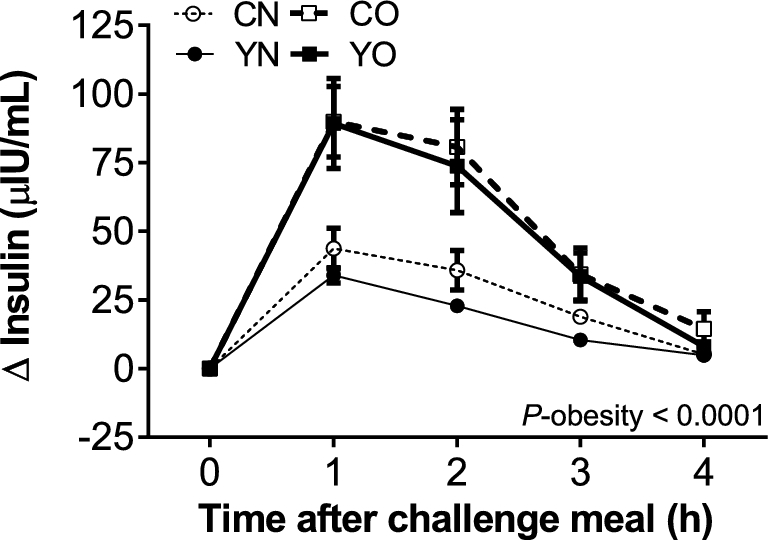
Incremental changes in postprandial plasma insulin in healthy obese and nonobese premenopausal women after consuming either low-fat yogurt or control food followed by a challenge meal at week 9 of the intervention. Data are means ± SEMs, *n* = 30. The effects of obesity status (obese compared with nonobese), dietary treatment (low-fat yogurt compared with control food), and the obesity × treatment interaction on net iAUC were determined by 2-factor ANOVA (PROC GLM). CN, control nonobese; CO, control obese; iAUC, incremental AUC; YN, yogurt nonobese; YO, yogurt obese; Δ, difference.

### Postprandial dysmetabolism

Postprandial TGs increased after the first challenge meal (Supplemental Figure 1B). At the first challenge meal, obese TG net iAUC was higher than in nonobese participants (Table 2, *P_ _*= 0.0027), but unchanged by the intervention (*P* = 0.60). Additional length of the intervention had no effect on postprandial TGs, as the AUCs were similar after the second challenge meal (Table 3).

Control obese (CO) plasma glucose was higher at 1 h after the first challenge meal, whereas YO glucose at 1 h was unchanged (Figure 1D). YN and control nonobese (CN) plasma glucose means were lower at 1 h after the first challenge meal. The net iAUCs for plasma glucose were different between groups (Table 2, *P*-interaction_ _= 0.0013). A subgroup analysis indicated that premeal yogurt consumption decreased postprandial hyperglycemia in obese participants and reduced postprandial hypoglycemia in the nonobese participants (*P* < 0.05). Additional yogurt consumption did not affect the postprandial response at the second challenge meal, as glucose ΔAUC (AUC_wk 9_ – AUC_wk 0_) was unchanged (Table 3, *P* = 0.43).

Postprandial plasma insulin was only determined at the second challenge meal because postprandial glucose exhibited similar responses at both time points. Postprandial insulin increased in all groups (Figure 2). The insulin net iAUC in obese participants was higher than in nonobese participants (*P_ _*< 0.0001), but unchanged by dietary treatment (*P* = 0.45).

## Discussion

Consumption of the challenge meal increased postprandial IL-6, depleted sCD14, and increased the LBP-to-sCD14 ratio. Postprandial LPS activity net iAUC increased to a similar extent as a prior report (20). In the present study, the PyroGene LPS activity C_max_ values of 16–20 EU/mL were higher than the typical C_max_ values of 0–5 EU/mL reported with the use of the *Limulus* Amebocyte Lysate assay. Baseline levels of PyroGene LPS activity of participants in the present study were 11–17 EU/mL (15). Thus, the increased LPS activity C_max_ in the present study is a result of relatively high baseline values detected by the PyroGene method. The postprandial endotoxemia response depends on obesity status, lipid amount, and lipid emulsification (21, 22). In obese men, postprandial LPS activity from 10 or 40 g fat was positively associated with chylomicron-rich TGs, but this relation was not observed in normal-weight men (21).

sCD14 transfers cell-bound immunocyte LPS to lipoproteins, thereby inhibiting proinflammatory cytokine production (23). LBP is associated with proinflammatory LPS actions (21, 24). Lower postprandial LBP-to-sCD14 ratios suggest improvement in intestinal barrier function and reduced endotoxin bioactivity. For example, an increased plasma LBP-to-sCD14 ratio was associated with inflammation and higher bioactivity of endotoxin in rodents (25). Also, increased postprandial LBP-to-sCD14 ratios were positively associated with IL-6 in men who consumed a mixed-fat challenge meal providing 33 g of fat (26).

In the same cohort, consumption of 339 g (12 oz) low-fat yogurt/d for 9 wk improved fasting plasma biomarkers of inflammation and endotoxin exposure in obese and nonobese women (15). Premeal consumption of 226 g (8 oz) yogurt acutely inhibited postprandial sCD14 loss in obese participants, relative to the controls. However, chronic yogurt consumption did not further improve the postprandial sCD14 response at week 9 of the study, or change baseline sCD14 concentrations. The postprandial ΔLBP-to-sCD14 AUC at week 9 was reduced by yogurt consumption, benefiting nonobese and obese individuals. However, this change was not sufficient to reduce postprandial IL-6 AUC beyond the first challenge meal.

Reduced endotoxin bioactivity may partly explain the mechanism by which premeal yogurt consumption inhibits postprandial IL-6. Endotoxins are potent proinflammatory molecules (8, 27). Circulating IL-6 increases postprandially within 4 h of high-calorie mixed-fat challenge meals (16, 28, 29). Increased postprandial LBP-to-sCD14 after a mixed-fat challenge meal was associated with IL-6 in healthy men, indicating that LPS handling shifts to more proinflammatory outcomes (26). Other classical inflammatory biomarkers such as TNF-α and high-sensitivity C-reactive protein do not increase postprandially, possibly due to their delayed response (28, 30–33).

A reduction in postprandial hyperglycemia may partly explain how yogurt inhibited postprandial IL-6 in obese women. Postprandial glucose induces oxidative stress (1, 34). Oxidative stress consequently stimulates inflammation by increasing mitogen-activated protein kinase and NF-κB signaling (5).

The present study is consistent with prior observations that obese individuals have dysregulated postprandial metabolism and insulin resistance (35). Premeal low-fat yogurt consumption might improve postprandial metabolism by several mechanisms. Dairy calcium diminishes postprandial lipidemia induced by consumption of a mixed meal, possibly via preventing intestinal absorption of some SFAs by forming calcium soaps (11). However, this does not appear to be a primary mechanism in the present study, as the intervention did not affect postprandial TGs. Thus, the impact of calcium reformulation of the soy pudding during the intervention was minimal. Dairy proteins have previously improved postprandial metabolism after consuming challenge meals consisting of 80 g butterfat with bread or pizza (10, 36). Alternatively, consumption of yogurt or dairy protein may decrease the gastric emptying rate (36, 37). Gastric emptying rate affects the magnitude and timing of postprandial glucose and insulin response by influencing the absorption of ingested nutrients (38). Rapid gastric emptying is also associated with postprandial hypoglycemia induced by excessive insulin release (39). However, we did not observe a difference in insulin secretion in either obese or nonobese individuals. An alternative mechanism for glycemic modulation by yogurt might exist and needs further investigation. The response of additional glucoregulatory hormones such as glucagon or glucagon-like peptide-1 should be considered, as well as a potential inhibition of dipeptidyl peptidase 4 activity by yogurt peptides.

A limitation of this study is that only healthy, premenopausal female participants were included to increase the homogeneity of responses. Also, the timing of plasma collection was not optimal for all biomarkers. A postprandial glucose peak was not observed in YO, CN, or YN. It is plausible that the glucose peak occurred between 0 and 1 h and was not captured by the selected study time points. In a postprandial study in normal-weight participants with a challenge meal similar to the present study, glucose peaked at 30 min and returned to baseline at 60 min (40). Also, several other factors not controlled for in the study design can affect postprandial health (41). Therefore, it remains to be determined whether such beneficial effects apply to male individuals and individuals with metabolic abnormalities.

The low-fat, sweetened yogurt was selected for the intervention because of the wide availability of this food in the US market, whereas the soy pudding was a nondairy, nonfermented control snack with similar macronutrient content. It should be noted that some ingredients in the test foods were different. Yogurt contained gelatin and pectin, and the low-fat soy pudding contained locust bean gum, pectin, and soy lecithin. Recent studies conducted in rodents have identified differences in the postprandial lipemia after consumption of emulsified soy or milk polar lipids (42, 43). In mice fed high-fat diets, consumption of emulsifiers in drinking water (1%, wt:vol) for 12 wk increased fat mass, fecal LPS activity, and gut barrier dysfunction (44). In the present study, it is unlikely that soy lecithin significantly affected the postprandial response, as consumption of low-fat yogurt and soy pudding led to similar postprandial plasma triacylglycerol and LPS activity responses.

The 2015–2020 Dietary Guidelines for Americans advise that low-fat dairy, including yogurt, should be included in a healthy eating pattern (45). The present study supports this recommendation, in that premeal low-fat yogurt consumption improved the postprandial glucose response and markers of inflammation and metabolic endotoxemia. However, the yogurt supplied 17 g added sugars/serving of dairy, which should be limited to 10% of calories/d. Therefore, consumers seeking to increase yogurt intake should be advised to maintain a healthful eating pattern.

In conclusion, premeal consumption of 226 g (8 oz) of yogurt improved acute postprandial dysfunction associated with a high-fat, high-calorie challenge meal in obese and nonobese women. Premeal yogurt consumption inhibited postprandial IL-6 and improved LBP-to-sCD14 ratio and glucose metabolism in both obese and nonobese participants. Daily consumption of yogurt for 9 wk further improved LBP-to-sCD14 ratios, but not postprandial IL-6. This suggests that daily consumption of yogurt may have a moderate long-term benefit in relation to metabolic endotoxemia, but the duration of 9 wk was insufficient to further reduce postprandial inflammation. Thus, premeal yogurt consumption is a feasible strategy to improve postprandial metabolism in apparently healthy nonobese and obese individuals.

## Supplementary Material

Supplemental data

## Notes

Supported by the National Dairy Council.

Author disclosures: RP, DMD, KKP, DAM, CC, RSB, and BWB, no conflicts of interest.

The funding agency facilitated peer review of the original research proposal, and feedback from the review process was incorporated into the final study design. The funding agency had no other involvement in the design, implementation, analysis, interpretation, or publication of data.

Supplemental Methods, Supplemental Tables 1 and 2, and Supplemental Figures 1 and 2 are available from the “Supplementary data” link in the online posting of the article and from the same link in the online table of contents at https://academic.oup.com/jn/.

Abbreviations used: C_max_, maximum concentration; CN, control nonobese; CO, control obese; EU, endotoxin units; iAUC, incremental AUC; LBP, LPS-binding protein; sCD14, soluble CD14; YN, yogurt nonobese; YO, yogurt obese; Δ, difference.
