# Supplementary material for: Premeal Low-Fat Yogurt Consumption Reduces Postprandial Inflammation and Markers of Endotoxin Exposure in Healthy Premenopausal Women in a Randomized Controlled Trial
Source: J Nutr. 2018 May 15;148(6):910–6. doi: 10.1093/jn/nxy046 (PMC5991203; doi:10.1093/jn/nxy046)
Supplement: Supplemental data [file nxy046_supplemental_files.docx]

**Supplemental Methods**

*Analysis of isoflavones in soy pudding by HPLC.* Hydrochloric acid (HCl) (37%) was from Acros Organics (Morris Plains, NJ). Daidzein, glycitein, genistein, butylated hydroxytoluene (BHT), thiobarbituric acid (TBA), trichloroacetic acid (TCA) and 1,1,3,3-tetramethoxypropane (TMP) were from Sigma-Aldrich (St. Louis, MO). Ethanol, acetic acid, acetonitrile, sodium acetate, sodium hydroxide, and isobutyl alcohol were from Fisher Scientific (Fairlawn, NJ). Soy puddings of chocolate, vanilla, chocolate/vanilla swirl flavor were from ZenSoy (South Hackensack, NJ).

Isoflavones were extracted according to Franke et al. with modifications (1). Briefly, 1 g soy pudding was added to 6.25 mL of 37% HCl and 30 mL of 96% ethanol (containing 0.05% BHT as antioxidant). This slurry was vortexed for 10 s followed by 10 min of sonication at room temperature (Fisher Scientific, Pittsburg, PA). After refluxing the mixture for 2 h at 90 °C, the mixture was cooled to room temperature and the ethanol lost during refluxing was replaced. Then, 1.2 mL of the above mixture was removed and centrifuged at 12,000 × g for 10 min.

Isoflavones were analyzed by HPLC as previously described, with minor modifications (1). A Shimadzu LC-20XR system was equipped with an SPD-20AV detector set to 260 nm. A 10 μL injection was eluted through a 250 mm × 4.6 mm i.d., 5 μm Waters Xterra MS C_18_ column (Waters Corporation, Milford, MA) via a binary gradient of acetic acid/water (10/90 V/V) (A) and acetonitrile (B). The proportion of B increased from 23 to 70% over 8 min, then decreased to 23% until 9 min, and held at 23% until 21 min. Isoflavones were quantified using the calibration curve.

**Supplemental Table 1.** Nutrient comparison of low-fat yogurt and the soy pudding control food

| **Nutrients per serving** | **Low-fat yogurt**^1^ | **Control food**^2^ |
| --- | --- | --- |
| Serving size (g) | 113 | 108 |
| Energy (kcal) | 110 | 110 |
| Total Fat (g) | 1 | 1 |
| Carbohydrate (g) | 22 | 22 |
| Sugars (g) | 17 | 17 |
| Protein (g) | 3 | 2-3^3^ |
| Cholesterol (mg) | 5 | 0 |
| Sodium (mg) | 60 | 55-70^4^ |
| Calcium (mg) | 150 | 60-150^4^ |
| Vitamin A (IU) | 500 | 200 |
| Vitamin D (μg) | 2.25 | 2.25 |

^1^Ingredients: Cultured pasteurized grade A low fat milk, sugar, strawberries (or banana puree/ peaches/raspberries, depending on the flavors), modified corn starch, nonfat milk, Kosher gelatin, citric acid, tricalcium phosphate, colored with carmine, natural flavor, pectin, retinol acetate, cholecalciferol. Contained disclaimer that product meets National Yogurt Association criteria for live and active culture yogurt (Contains *Lactobacillus bulgaricus* and *Streptococcus thermophilus* with at least 10^7^ cultures per g at manufacture).

^2^Ingredients: Filtered water, organic soymilk, organic granulated can sugar, organic corn starch, organic cocoa (processed with alkali) or organic vanilla extract, natural flavors, carrageenan, calcium carbonate, evaporated salt, organic soy lecithin, organic locust bean gum, retinyl palmitate, ergocalciferol, riboflavin, cyanocobalamin.

^3^Different flavors of pudding contained either 2 or 3 g of protein per serving.

^4^Due to the reformulation by the manufacture during the intervention, the sodium content decreased from 70 to 55 mg per serving and the calcium decreased from 150 to 60 mg per serving (n = 27, on the old formulation; n = 37, on the new formulation).

**Supplemental Table 2.** Postprandial LPS activity C_max_ values in healthy premenopausal women after consuming either low-fat yogurt or control food followed by a challenge meal at wk 0 of the intervention.^1^

| C_max_ | **Group** | | | | **Significance** (*P*) | | |
| --- | --- | --- | --- | --- | --- | --- | --- |
|  | CN | YN | CO | YO | Obesity | Treatment | Interaction |
| LPS activity (EU/mL) | 17.7 ± 1.31 | 15.6 ± 0.930 | 20.1 ± 1.27 | 17.2 ± 0.970 | 0.97 | 0.85 | 0.68 |

^1^Data are means ± SEMs, n = 30. EU, endotoxin units. The effects of obesity status (*obese vs. non-obese*), dietary treatment (*low-fat yogurt vs. control food*), and the obesity × treatment interaction on net iAUC were determined by Two-way ANOVA (PROC GLM).

**
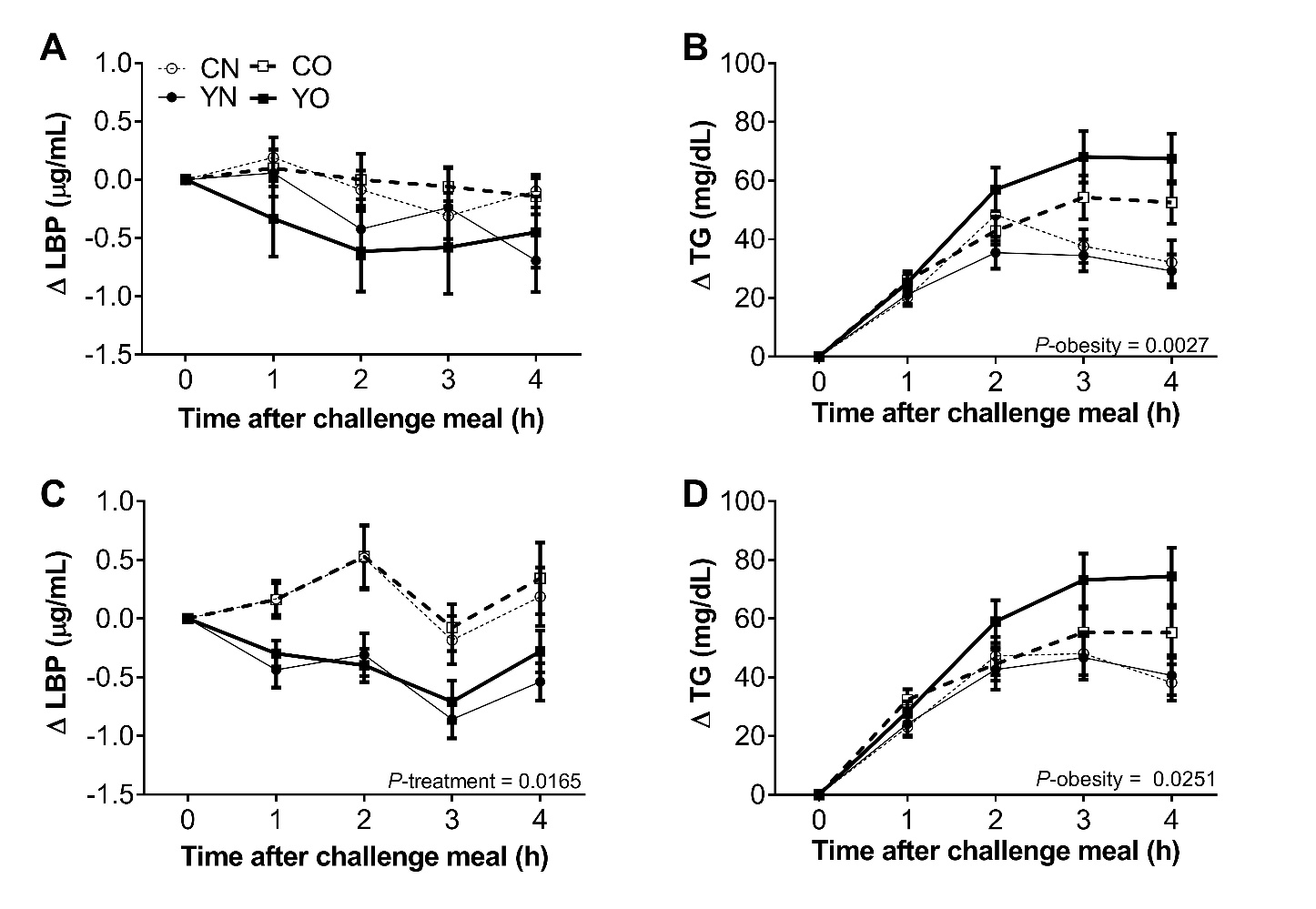
**

**Supplemental Figure 1** Incremental postprandial changes in plasma LBP (A) and triglycerides (B) at week 0 of the intervention; plasma LBP (C) and triglycerides (D) at week 9 of the intervention in healthy premenopausal women that consumed low-fat yogurt or the control food followed by the challenge meal. CN, control non-obese; CO, control obese; YN, yogurt non-obese; YO, yogurt obese. LBP, lipopolysaccharide-binding protein. Data are means ± SEMs, n = 30. The effects of obesity status (*obese vs. non-obese*), dietary treatment (*low-fat yogurt vs. control food*), and the obesity × treatment interaction on net iAUC were determined by Two-way ANOVA (PROC GLM).

**
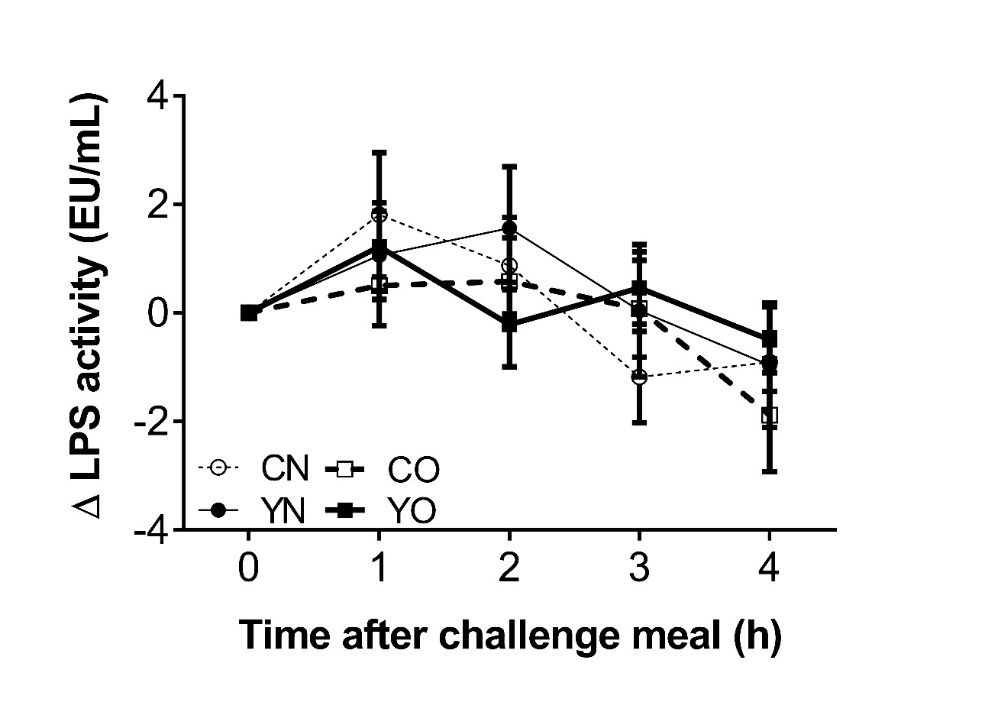
**

**Supplemental Figure 2** Incremental changes in postprandial plasma LPS activity in healthy premenopausal women after consuming either low-fat yogurt or control food followed by a challenge meal at week 9 of the intervention. EU, endotoxin units; CN, control non-obese; CO, control obese; YN, yogurt non-obese; YO, yogurt obese; data are means ± SEMs, n = 30. The effects of obesity status (*obese vs. non-obese*), dietary treatment (*low-fat yogurt vs. control food*), and the obesity × treatment interaction on net iAUC were determined by Two-way ANOVA (PROC GLM).

**Supplemental References**

1. Franke AA, Custer LJ, Cerna CM, Narala KK. Quantitation of phytoestrogens in legumes by HPLC. J Agric Food Chem. 1994;42:1905-13.
